# Evaluation of Automated Treatment Planning and Organ Dose Prediction for Lung Stereotactic Body Radiotherapy

**DOI:** 10.7759/cureus.18473

**Published:** 2021-10-04

**Authors:** Zi Ouyang, Tingliang Zhuang, Gaurav Marwaha, Matthew D Kolar, Peng Qi, Gregory M Videtic, Kevin L Stephans, Ping Xia

**Affiliations:** 1 Department of Radiation Oncology, Cleveland Clinic, Cleveland, USA

**Keywords:** treatment planning, rtog 0813, dose prediction, autoplan, sbrt

## Abstract

Purposes: To evaluate whether the auto-planning (AP) module can achieve clinically acceptable treatment plans for lung stereotactic body radiotherapy (SBRT) and to evaluate the effectiveness of a dose prediction model.

Methods: Twenty lung SBRT cases planned manually with 50 Gy in five fractions were replanned using the Pinnacle (Philips Radiation Oncology Systems, Fitchburg, WI) AP module according to the dose constraint tables from the Radiation Therapy Oncology Group (RTOG) 0813 protocol. Doses to the organs at risk (OAR) were compared between the manual and AP plans. Using a dose prediction model from a commercial product, PlanIQ (Sun Nuclear Corporation, Melbourne, FL), we also compared OAR doses from AP plans with predicted doses.

Results: All manual and AP plans achieved clinically required dose coverage to the target volumes. The AP plans achieved equal or better OAR sparing when compared to the manual plans, most noticeable in the maximum doses of the spinal cord, ipsilateral brachial plexus, esophagus, and trachea. Predicted doses to the heart, esophagus, and trachea were highly correlated with the doses of these OARs from the AP plans with the highest correlation coefficient of 0.911, 0.823, and 0.803, respectively.

Conclusion: Auto-planning for lung SBRT improved OAR sparing while keeping the same dose coverage to the tumor. The dose prediction model can provide useful planning dose guidance.

## Introduction

Beyond accuracy in dose calculation, computer-aided optimization and automation aim to improve planning efficiency, consistency, and quality. Tools such as auto-planning, RapidPlan (Varian Medical Systems, Palo Alto, CA), and multi-criteria optimization (MCO) are developed by different vendors and implemented for clinical use. Large variations in plan quality are observed in multi-institutional studies [1], which imply that plan quality may be limited by planners’ experience and expertise. With increased automation in treatment planning, the resultant plan quality is less dependent on the user experience, while the planning efficiency and consistency can be improved.

Auto-planning (AP) is an integrated module in the Pinnacle (Philips Radiation Oncology Systems, Fitchburg, WI) treatment planning system. It mimics the iterative manual planning process to achieve the prescription doses and spare organs at risk (OARs) [2]. Studies have confirmed that the AP module produced clinically acceptable treatment plans for the brain, head, neck, esophagus, lung, and prostate [3-10].

The utilization of lung stereotactic body radiotherapy (SBRT) has increased steadily over the past two decades [11-17]. SBRT with a minimum biological equivalent dose (BED) of 100 Gy has been shown to be safe and effective at curing stage I non-small-cell lung cancer in medically inoperable patients [15]. At such high BED and fractional doses, the standardization of treatment planning is critical to the treatment quality. To maintain consistent plan quality, many institutions have implemented a peer-review process for SBRT plans, adopting a dosimetry audit process used in multicenter clinical trials [18]. The challenge at the time of a plan quality peer review or audit is that it is only as good as the dosimetric metrics that are established for a specific disease presentation and is not patient-specific. Knowledge-based models and artificial intelligence (AI)-based predictions are being introduced to address this challenge, but practical solutions are not readily available for general planners and medical physicists. In planning radiotherapy for some cancers, for example, in the head and neck, the number of OARs also makes knowledge or AI-based predictions challenging due to the possible trade-off among these OARs. When planning lung SBRT cases, however, the number of OARs is relatively modest and the relationship between them is less complex. This makes consideration of potential OAR trade-offs simpler. For these reasons, planning automation and accurate plan quality prediction may be easier to achieve. The present study seeks to evaluate the AP module in planning for lung SBRT and to compare its dosimetric results to the plan predictions from a commercially available product. These dose predictions potentially will serve two purposes: (a) to provide input objectives to the AP module and (b) to validate the quality of AP plans.

## Materials and methods

Twenty patients treated with definitive, manually-planned intensity-modulated radiation therapy (IMRT) or volumetric modulated arc therapy (VMAT) for lung SBRT at our institution from 2014 to 2015 were randomly selected and replanned with the AP module without manual adjusting. The AP module mimics the manual planning process, separates overlapped contours, creates tuning structures, adjusts hot and cold spots, and optimizes the plan iteratively [8]. Both the original manual plans and AP replans were prescribed with 50 Gy in five fractions according to the Radiation Therapy Oncology Group (RTOG) 0813 protocol and planned using the Pinnacle treatment planning system (version 9.10, Philips Radiation Oncology Systems, Fitchburg, WI). A set of AP parameters were determined based on the RTOG 0813 dose constraints and phantom testing. The AP parameter settings and the planning objectives for the central and peripheral tumors are listed in Tables 1, 2. Among the planning structures, “Ring” was a 4-cm expansion of the planned target volume (PTV) minus the 2-cm expansion. The AP replans without further manual adjustment were qualitatively judged by a physician based on the conformality, sharpness of dose fall-off (isodose lines at 2 cm beyond the edge of the PTV), and verification using RTOG 0813 constraints (Table 3). Time from initiating the AP process to arrive at an acceptable plan was recorded.

**Table 1:** AP advanced settings. AP: auto-planning; ROI: region of interest.

Max iterations	50
Engine type	Biological
Tuning balance	20%
Dose fall-off margin	1.5 cm
Hot-spot maximum goal	150%
Use cold-spot ROIs	No

**Table 2:** AP planning objectives. AP: auto-planning; PBT: proximal bronchial tree; Cord: spinal cord; DVH: dose-volume histogram.

Target location	Structure	Type	Primary goal		Priority	Compromise
			Dose (cGy)	Volume (%)		
Central	Heart	Max dose	5150	-	High	Checked
	Heart	Max DVH	3100	1.5	High	Checked
	Cord	Max dose	1400	-	High	Checked
	Esophagus	Max dose	5150	-	High	Checked
	Esophagus	Max DVH	2650	10	High	Checked
	PBT	Max dose	5150	-	High	Checked
	PBT	Max DVH	1700	20	High	Checked
	Trachea	Max dose	5150	-	High	Checked
	Trachea	Max DVH	1700	20	High	Checked
	Whole lung	Max DVH	1900	10	High	Checked
	Ring	Max dose	3500	-	High	Checked
Peripheral	Ring	Max dose	2500	-	High	Checked

**Table 3:** Our institutional scorecard for the evaluation of 50 Gy in five fraction lung SBRT plans. Contours existing in the plan are evaluated and the results show “Met” or “Not Met,” indicating whether the planning goals are met. SBRT: stereotactic body radiotherapy; PTV: planned target volume; PBT: proximal bronchial tree; IPSI BP: ipsilateral brachial plexus; DVH: dose-volume histogram

Structure	Type	Primary goal		Primary achieved		Result
		Dose (cGy)	Volume	Dose (cGy)	Volume	
PTV	Min DVH	5000	95%			
PTV	Min DVH	4500	99%			
IPSI BP	Max DVH	3000	3 cm^3^			
IPSI BP	Min dose	3200				
Heart	Mean DVH	3200	15 cm^3^			
Heart	Max dose	5250				
Trachea	Max DVH	1800	4 cm^3^			
Trachea	Max dose	5250				
PBT	Max DVH	2750	4 cm^3^			
PBT	Max dose	5250				
Esophagus	Max DVH	2750	5 cm^3^			
Esophagus	Max dose	5250				
Whole lung	Max DVH	2000	10%			
Cord	Max DVH	2250	0.25 cm^3^			
Cord	Max DVH	1350	0.5 cm^3^			
Heart	Max dose	3000				

All manual and AP plans used Novalis Tx (Varian Medical Systems, Palo Alto, CA) or Edge machines (BrainLab, Munich, Germany) equipped with high definition multileaf collimators. Beam energy was 6 MV flat stereotactic radiosurgery (SRS) for Novalis Tx and 6 MV flattening-filter-free (FFF) for Edge. The AP advanced settings focused on target conformality and allowed hot spots within the target. The following dosimetric parameters were compared between planning strategies: PTV coverage; conformality index (CI); whole lung V_20%_; and maximum point doses (D_max_) to the spinal cord, esophagus, heart, trachea, proximal bronchial tree (PBT), and ipsilateral brachial plexus (ipsi BP).

The PlanIQ (Sun Nuclear Corporation, Melbourne, FL) is a commercial product that predicts possible OAR sparing or feasibility. The targets are assumed to have 100% of uniform prescription dose coverage, which is not used for dose coverage prediction and appears clinically impossible. The OAR sparing prediction and the dose fall-off outside the targets are calculated using the heterogeneous patient dataset, taking into account the high (penumbra-driven) and low (percent depth dose [PDD] and scatter-driven) gradient dose spreading [19]. The PlanIQ predictions are assigned with f-values (feasibility factor), which indicate the feasibility in achieving the predicted OAR sparing. The f-value ranges from 0 (unachievable) to 1 (easily achievable). The AP dosimetric endpoints were compared with the predicted values from PlanIQ, and correlations between the AP doses and predictions were tested.

The Mann-Whitney U test [20] was used to compare the AP planning time between central and peripheral tumors and between VMAT and IMRT techniques. The Wilcoxon signed-rank test [21] was used to compare the PTV coverage and OAR sparing for each pair of manual and AP plans. Spearman’s rank correlation [22] was used to describe the correlation between the PlanIQ predictions and AP plans.

## Results

**Figure 1:**
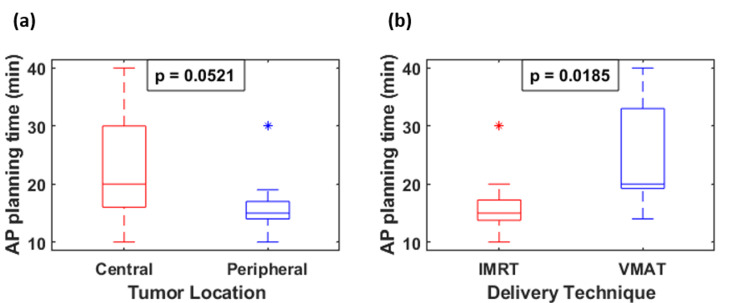
Comparison of AP time for tumor locations and delivery techniques. P-values are calculated using the Mann–Whitney U test. VMAT: volumetric modulated arc therapy; IMRT: intensity-modulated radiation therapy; AP: auto-planning.

Of the 20 patients, 10 had central and the other 10 had peripheral tumors. The median tumor size was 3.55 cm (range: 0.9-6.8 cm, Table 4). The median time for the AP treatment planning process was 17 minutes per plan (range: 10-40). As shown in Figure 1, the median time for AP for central vs. peripheral tumors was 20 vs. 15 minutes (p = 0.0521), and for IMRT vs. VMAT was 15 vs. 20 minutes (p = 0.0185). The quality of AP vs. manual plans was “better” in 15%, “equally acceptable” in 80%, and “worse” in 5% per physician judgment based on the target coverage, OAR sparing, and three-dimensional isodose distributions.

**Table 4:** Tumor location, size, and treatment delivery type for the 20 patients. VMAT: volumetric modulated arc therapy; IMRT: intensity-modulated radiation therapy.

Patient No.	Tumor location	Size (cm)	Delivery type
1	Central	5.6	VMAT
2	Central	5.9	IMRT
3	Central	6.8	IMRT
4	Central	5.3	VMAT
5	Central	2.4	IMRT
6	Peripheral	2.3	IMRT
7	Central	3.3	IMRT
8	Central	4.2	VMAT
9	Peripheral	3.8	IMRT
10	Central	4.4	VMAT
11	Central	0.9	VMAT
12	Peripheral	2.5	IMRT
13	Central	3.8	IMRT
14	Peripheral	1.8	IMRT
15	Peripheral	1.6	VMAT
16	Peripheral	4.5	IMRT
17	Peripheral	3.3	IMRT
18	Peripheral	1.0	IMRT
19	Peripheral	1.9	VMAT
20	Peripheral	3.9	IMRT

**Figure 2:**
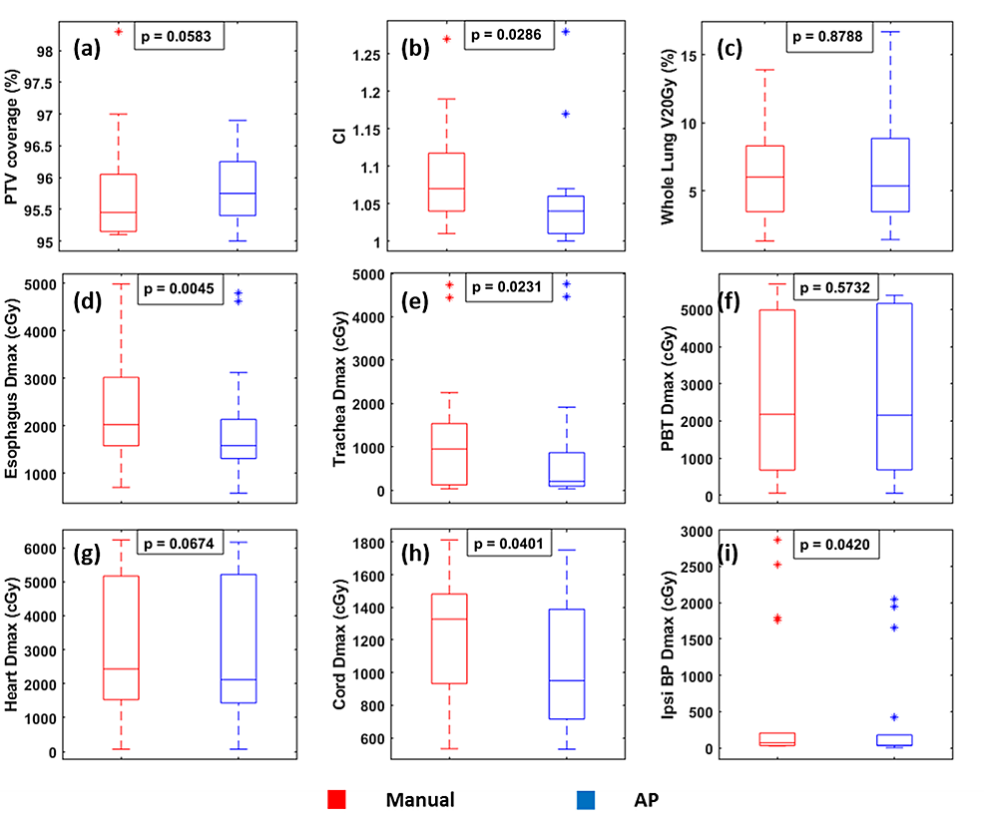
Dosimetric comparison between the manual (red) and AP (blue) plans. Median, interquartile range, minimum, and maximum values are shown in each figure. Outliers are marked with “*”. P-values are calculated using the Wilcoxon signed-rank test. AP: auto-planning; PTV: planned target volume; PBT: proximal bronchial tree; ipsi BP: ipsilateral brachial plexus; CI: conformality index.

Figure 2 depicts the dosimetric comparison between the AP and manual plans. All AP and manual plans achieved clinically required target coverage; at least 95% of the PTV received 100% of the prescription dose. The median values for the variables compared between manual and AP plans are listed in Table 5.

**Table 5:** Comparison of dosimetry variables among manual and AP plans. PTV: planned target volume; PBT: proximal bronchial tree; ipsi BP: ipsilateral brachial plexus; AP: auto-planning; CI: conformality index.

OARs	Manual plans	AP plans	P-values
V_50Gy_ of PTV	95.5%	95.8%	0.0583
CI	1.07	1.04	0.0286*
V_20Gy_ of whole lung	6%	5.4%	0.8788
D_max_ esophagus	20.3 Gy	15.9 Gy	0.0045*
D_max_ trachea	9.5 Gy	2.0 Gy	0.0231*
Dmax PBT	21.8 Gy	21.5 Gy	0.5732
Dmax heart	24.3 Gy	21.1 Gy	0.0674
D_max_ spinal cord	13.3 Gy	9.5 Gy	0.04*
D_max_ ipsi BP	0.71 Gy	0.37 Gy	0.042*

**Figure 3:**
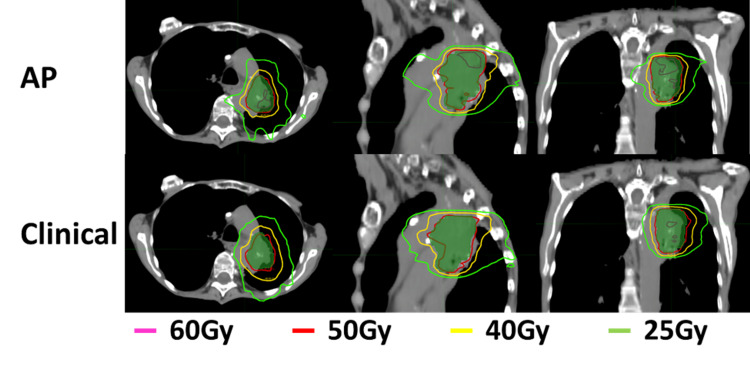
An example of an isodose distribution of AP compared to the clinical plan. The green color wash shows the PTV. AP: auto-planning; PTV: planned target volume.

**Figure 4:**
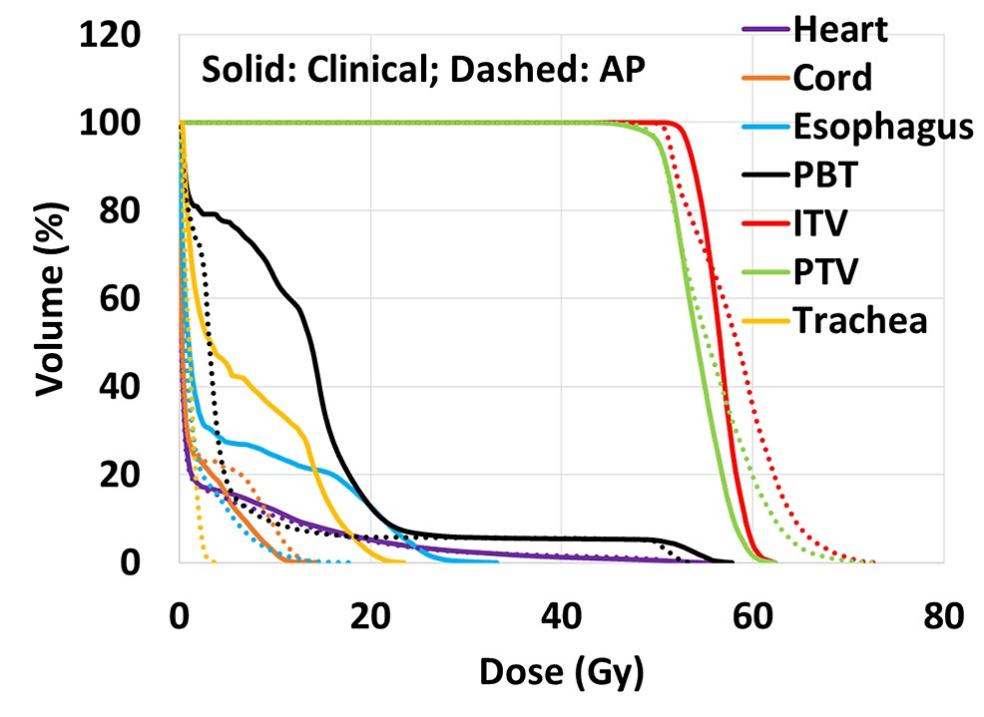
DVHs of the AP and clinical plans for the target volumes, heart, spinal cord, esophagus, trachea, and PBT. Solid lines represent the clinical plan and dashed lines represent the AP plan. DVH: dose-volume histogram; AP: auto-planning; PBT: proximal bronchial tree; ITV: internal target volume; PTV: planned target volume.

Figures 3, 4 show the isodose distributions and dose-volume histograms (DVHs) of the comparison between the AP and clinical plans for one patient. The AP plan had higher maximum doses within the PTV and generally lower doses to the OARs. The isodose distribution also showed that the dose fall-off was steeper in the AP plan.

**Figure 5:**
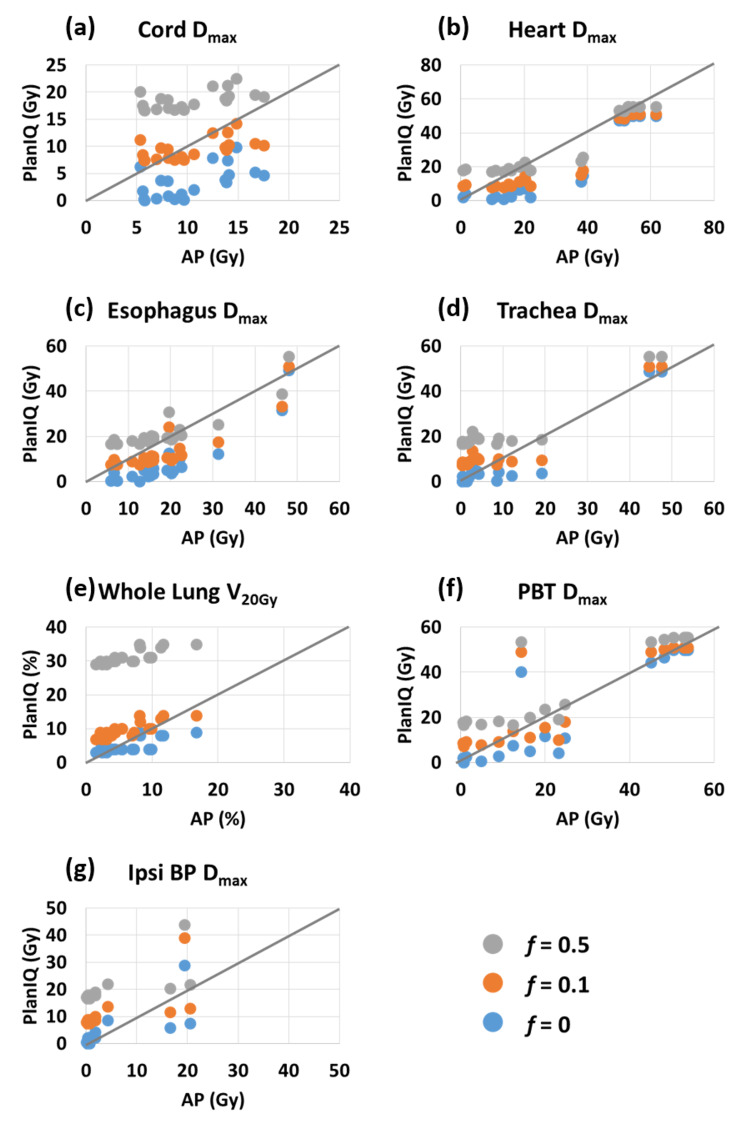
PlanIQ predictions plotted against AP dosimetric values. Blue: f = 0; orange: f = 0.1; and gray: f = 0.5. Increasing f factor indicates higher feasibility in achieving predicted OAR sparing. A reference line y = x is shown in each figure. Points following the reference line well show strong correlations. AP: auto-planning; OAR: organ at risk; PBT: proximal bronchial tree; ipsi BP: ipsilateral brachial plexus.

The dose endpoints for OARs were compared between the AP plans and PlanIQ predictions with the feasibility factor, f, set to 0, 0.1, and 0.5, respectively. Figure 5 shows the PlanIQ predictions plotted against the AP parameters with a reference line y = x. The reference line represents the ideal prediction. The correlation between the PlanIQ and AP dosimetric parameters was tested using the Spearman rank-order correlation (Table 6). The spinal cord Dmax had a moderate correlation, while other OARs had high correlations.

**Table 6:** Spearman’s rank-order correlation between the predicted and AP OAR endpoints. High correlations are observed for Dmax of the heart, esophagus, and trachea, and V_20Gy_ of the whole lung, while moderate correlation is observed for Dmax of the spinal cord. AP: auto-planning; OAR: organ at risk; PBT: proximal bronchial tree; Ipsi BP: ipsilateral brachial plexus.

Spearman correlation	f = 0	f = 0.1	f = 0.5
Cord D_max_	0.5489	0.5534	0.5534
Heart D_max_	0.8909	0.9106	0.9034
Esophagus D_max_	0.8229	0.8229	0.8229
Trachea D_max_	0.8027	0.7846	0.7842
Whole lung V_20Gy_	0.7780	0.8216	0.7729
PBT D_max_	0.9426	0.9380	0.9227
Ipsi BP D_max_	0.7861	0.7861	0.7861

## Discussion

The AP treatment planning time for IMRT was significantly shorter than that for VMAT, which is similar to our experience with manual planning. Step-and-shoot IMRT plans typically have fewer control points thus shorter calculation time compared to that of VMAT plans, and gantry speed is not a constraint for IMRT optimization. While the difference was not significant, the AP treatment planning time was shorter for peripheral targets than that for central targets, which appears intuitive as central targets are closer to more OARs. AP treatment planning time also depended on other factors, such as tumor size, computational power, dose grid size, and resolution. While the total planning time was not compared between manual and AP plans, the AP plan quality achieved clinical acceptance with minimal human intervention, thus saving planners’ time and improving efficiency. Creemers et al. have noted that AP reduces the planners’ “hands-on time” by 75% [3]. In clinical treatment planning, efficiency and quality are not independent. Planners often work on multiple plans simultaneously with given deadlines. After meeting clinical acceptance criteria, further manual optimization may not be feasible due to the limit in time and resources. By reducing the “hands-on time,” better plan quality may also be achieved.

A recent study [23] by Lu et al. compared plan quality for four sites using three different advanced planning tools including AP. They showed that AP could improve plan quality, but the statistical power was limited by the small sample size-five patients for each site. Our study showed that the AP plans maintained the PTV coverage and significantly improved CI. Doses were also reduced in AP plans for all seven OARs, and four of the seven reached statistical significance (p < 0.05).

While AP mimics planners to progressively optimize IMRT and VMAT plans, the process is not closed-loop automation. The PlanIQ is designed to predict feasible DVHs to help guide the AP set-up; it also provides an initial plan quality check when the AP process finishes. Unlike knowledge-based planning, the PlanIQ uses a geometric relationship between the target and OARs and calculates the feasible DVHs without any dependence on prior treatment planning knowledge. It avoids the potential propagation of skewed data. In this work, the AP results and PlanIQ predictions had strong correlations for six of seven OARs, which indicated that PlanIQ could be used as an AP plan quality checker. Other studies have also shown that using PlanIQ predictions as planning guidance could improve plan quality [24].

## Conclusions

Auto-planning in lung SBRT improved OAR sparing while keeping the same dose coverage to the tumor. Of tested AP replans, 95% were at least equally acceptable compared to the manual plans. The OAR dose predictions correlated strongly with the AP dosimetric endpoints on D_max_ of the heart, esophagus, trachea, PBT, and ipsilateral brachial plexus, as well as the whole lung V_20Gy_. AP is a reliable strategy to improve lung SBRT planning quality and efficiency, and the prediction tool may offer additional automation and quality assurance.

## References

[1] Das IJ, Cheng CW, Chopra KL, Mitra RK, Srivastava SP, Glatstein E (2008). Intensity-modulated radiation therapy dose prescription, recording, and delivery: patterns of variability among institutions and treatment planning systems. J Natl Cancer Inst.

[2] Hazell I, Bzdusek K, Kumar P (2016). Automatic planning of head and neck treatment plans. J Appl Clin Med Phys.

[3] Creemers IH, Kusters JM, van Kollenburg PG, Bouwmans LC, Schinagl DA, Bussink J (2019). Comparison of dose metrics between automated and manual radiotherapy planning for advanced stage non-small cell lung cancer with volumetric modulated arc therapy. Phys Imaging Radiat Oncol.

[4] Hansen CR, Nielsen M, Bertelsen AS (2017). Automatic treatment planning facilitates fast generation of high-quality treatment plans for esophageal cancer. Acta Oncol.

[5] Krayenbuehl J, Di Martino M, Guckenberger M, Andratschke N (2017). Improved plan quality with automated radiotherapy planning for whole brain with hippocampus sparing: a comparison to the RTOG 0933 trial. Radiat Oncol.

[6] Kusters JM, Bzdusek K, Kumar P (2017). Automated IMRT planning in Pinnacle: a study in head-and-neck cancer. Strahlenther Onkol.

[7] Nawa K, Haga A, Nomoto A, Sarmiento RA, Shiraishi K, Yamashita H, Nakagawa K (2017). Evaluation of a commercial automatic treatment planning system for prostate cancers. Med Dosim.

[8] Ouyang Z, Liu Shen Z, Murray E (2019). Evaluation of auto-planning in IMRT and VMAT for head and neck cancer. J Appl Clin Med Phys.

[9] Vanderstraeten B, Goddeeris B, Vandecasteele K, van Eijkeren M, De Wagter C, Lievens Y (2018). Automated instead of manual treatment planning? A plan comparison based on dose-volume statistics and clinical preference. Int J Radiat Oncol Biol Phys.

[10] Wang S, Zheng D, Zhang C (2017). Automatic planning on hippocampal avoidance whole-brain radiotherapy. Med Dosim.

[11] Bezjak A, Paulus R, Gaspar LE (2016). Efficacy and toxicity analysis of NRG Oncology/RTOG 0813 trial of stereotactic body radiation therapy (SBRT) for centrally located non-small cell lung cancer (NSCLC). Int J Radiat Oncol Biol Phys.

[12] Bezjak A, Paulus R, Gaspar LE (2016). Primary study endpoint analysis for NRG Oncology/RTOG 0813 trial of stereotactic body radiation therapy (SBRT) for centrally located non-small cell lung cancer (NSCLC). Int J Radiat Oncol Biol Phys.

[13] Okunieff P, Petersen AL, Philip A, Milano MT, Katz AW, Boros L, Schell MC (2006). Stereotactic body radiation therapy (SBRT) for lung metastases. Acta Oncol.

[14] Olsen JR, Robinson CG, El Naqa I (2011). Dose-response for stereotactic body radiotherapy in early-stage non-small-cell lung cancer. Int J Radiat Oncol Biol Phys.

[15] Onishi H, Shirato H, Nagata Y (2011). Stereotactic body radiotherapy (SBRT) for operable stage I non-small-cell lung cancer: can SBRT be comparable to surgery?. Int J Radiat Oncol Biol Phys.

[16] Stephans KL, Djemil T, Tendulkar RD, Robinson CG, Reddy CA, Videtic GM (2012). Prediction of chest wall toxicity from lung stereotactic body radiotherapy (SBRT). Int J Radiat Oncol Biol Phys.

[17] Taremi M, Hope A, Dahele M (2012). Stereotactic body radiotherapy for medically inoperable lung cancer: prospective, single-center study of 108 consecutive patients. Int J Radiat Oncol Biol Phys.

[18] Clark CH, Hurkmans CW, Kry SF (2017). The role of dosimetry audit in lung SBRT multi-centre clinical trials. Phys Med.

[19] Ahmed S, Nelms B, Gintz D, Caudell J, Zhang G, Moros EG, Feygelman V (2017). A method for a priori estimation of best feasible DVH for organs-at-risk: validation for head and neck VMAT planning. Med Phys.

[20] Mann HB, Whitney DR (1947). On a test of whether one of two random variables is stochastically larger than the other. Ann Math Stat.

[21] Wilcoxon F (1945). Individual comparisons by ranking methods. Biometrics Bulletin.

[22] Spearman C (1987). The proof and measurement of association between two things. Am J Psychol.

[23] Lu L, Sheng Y, Donaghue J, Liu Shen Z, Kolar M, Wu QJ, Xia P (2019). Three IMRT advanced planning tools: a multi-institutional side-by-side comparison. J Appl Clin Med Phys.

[24] Fried DV, Chera BS, Das SK (2017). Assessment of PlanIQ feasibility DVH for head and neck treatment planning. J Appl Clin Med Phys.

